# Identification of hub genes and transcription factor regulatory network for heart failure using RNA-seq data and robust rank aggregation analysis

**DOI:** 10.3389/fcvm.2022.916429

**Published:** 2022-10-28

**Authors:** Dingyuan Tu, Chaoqun Ma, ZhenYu Zeng, Qiang Xu, Zhifu Guo, Xiaowei Song, Xianxian Zhao

**Affiliations:** ^1^Department of Cardiology, Changhai Hospital, Naval Medical University, Shanghai, China; ^2^Department of Cardiology, Navy 905 Hospital, Naval Medical University, Shanghai, China

**Keywords:** heart failure, RNA-seq dataset, RUVSeq, robust rank aggregation, hub gene, biomarker, functional enrichment analysis, transcription factor

## Abstract

**Background:**

Heart failure (HF) is the end stage of various cardiovascular diseases with a high mortality rate. Novel diagnostic and therapeutic biomarkers for HF are urgently required. Our research aims to identify HF-related hub genes and regulatory networks using bioinformatics and validation assays.

**Methods:**

Using four RNA-seq datasets in the Gene Expression Omnibus (GEO) database, we screened differentially expressed genes (DEGs) of HF using Removal of Unwanted Variation from RNA-seq data (RUVSeq) and the robust rank aggregation (RRA) method. Then, hub genes were recognized using the STRING database and Cytoscape software with cytoHubba plug-in. Furthermore, reliable hub genes were validated by the GEO microarray datasets and quantitative reverse transcription polymerase chain reaction (qRT-PCR) using heart tissues from patients with HF and non-failing donors (NFDs). In addition, R packages “clusterProfiler” and “GSVA” were utilized for enrichment analysis. Moreover, the transcription factor (TF)–DEG regulatory network was constructed by Cytoscape and verified in a microarray dataset.

**Results:**

A total of 201 robust DEGs were identified in patients with HF and NFDs. STRING and Cytoscape analysis recognized six hub genes, among which *ASPN*, *COL1A1*, and *FMOD* were confirmed as reliable hub genes through microarray datasets and qRT-PCR validation. Functional analysis showed that the DEGs and hub genes were enriched in T-cell-mediated immune response and myocardial glucose metabolism, which were closely associated with myocardial fibrosis. In addition, the TF–DEG regulatory network was constructed, and 13 significant TF–DEG pairs were finally identified.

**Conclusion:**

Our study integrated different RNA-seq datasets using RUVSeq and the RRA method and identified *ASPN*, *COL1A1*, and *FMOD* as potential diagnostic biomarkers for HF. The results provide new insights into the underlying mechanisms and effective treatments of HF.

## Introduction

Heart failure (HF) is a complex clinical syndrome that results from dysfunction of ventricular filling or ejection, characterized by a variety of worsening symptoms and signs, including dyspnea, fatigue, and fluid retention (1). The occurrence of HF is predominantly caused by underlying myocardial diseases, while cardiac lesions from valves, vasculature, pericardium, heart rate/rhythm, or a combination of cardiac abnormalities may also result in cardiac malfunction (2). Despite the development of drug therapy and surgical interventional therapy, the morbidity and mortality of HF are increasing annually worldwide, which seriously threatens human health and quality of life (3, 4). Therefore, to improve the curative efficacy, it remains urgent to investigate the in-depth underlying molecular mechanisms of HF to facilitate its accurate diagnosis, early intervention, and precision therapy.

In recent years, the rapid progress of transcriptome sequencing technology provides new directions for the exploration of epigenetic changes and molecular mechanisms in different diseases, including neoplastic and non-neoplastic diseases (5, 6). Accordingly, an increasing volume of RNA sequencing (RNA-seq) and microarray datasets of HF has been uploaded in the Gene Expression Omnibus (GEO) database, providing opportunities for bioinformatics data mining of marker genes associated with HF (7). However, in comparison to cancer-related surgery, the number of heart transplantation surgeries is relatively small, which results in the small sample size and large batch effects of RNA sequencing or microarray datasets of HF. Therefore, to date, the bioinformatics data mining of HF still faces great challenges, especially regarding the integration and analysis of the RNA-seq data (RUVSeq) related to HF.

The robust rank aggregation (RRA) method, first proposed in 2012 by Kolde et al., is a rigorous approach using probabilistic models to analyze the significant probability of all elements in different sequencing or microarray datasets (8). Recently, the RRA algorithm has been extensively used to integrate data in different microarray platforms to screen the differentially expressed genes (DEGs) in multiple diseases, including thyroid carcinoma (9), prostate cancer (PCa) (10), and DCM (11). For example, Song et al. utilized the RRA method to integrate 10 eligible PCa microarray datasets from the GEO and identify four candidate biomarkers for prognosis of PCa (10). Ma et al. integrated four eligible dilated cardiomyopathy (DCM) microarray datasets from the GEO database and developed a 7-gene signature predictive of DCM by utilizing the RRA method (11). However, due to the greater difficulty in integrating sequencing data, the application of the previous RRA algorithm was limited to microarray data, and the RRA analysis of RUVSeq was still rarely reported. Removal of Unwanted Variation from RUVSeq, a Bioconductor package that generalizes a linear model to regress variance estimated from the expression of housekeeping genes, has been reported to be used to reduce batch effects due to different sequencing conditions (12), which provides a huge possibility for the combination of RUVSeq and the RRA method in integrating different RUVSeq sets and identifying HF-associated DEGs.

In the present study, RUVSeq and RRA analysis were performed for the first time based on four RNA-seq datasets in the GEO database to identify robust DEGs in HF samples and non-failing donor (NFD) samples, followed by Gene Ontology (GO) and Kyoto Encyclopedia of Genes and Genomes (KEGG) enrichment analysis for the DEGs. Moreover, three reliable HF-related hub genes with differential expression and excellent diagnostic efficiency, *ASPN*, *COL1A1*, and *FMOD*, were selected and validated using microarray datasets and human heart tissue assays. Gene set enrichment analysis (GSEA) and gene set variation analysis (GSVA) were further utilized to investigate potential functions of the hub genes. In addition, the transcription factor (TF)–DEG regulatory network was constructed based on the HF datasets and websites.

## Materials and methods

### Datasets search and inclusion criteria

The GEO database^1^ was searched to obtain the sequencing datasets based on the search terms of “heart failure” or/and “HF.” The search results and relevant datasets were filtered according to the following inclusion criteria: (i) the organism was filtered by “homo sapiens”; (ii) the study type was set as “expression profiling by high throughput sequencing”; (iii) RUVSeq for both HF samples and NFDs should be included in the dataset; (iv) the total number of samples should not be < 5; and (v) the raw data of the RNA-seq should be provided for reanalysis. Datasets that did not meet the aforementioned criteria were excluded. The selected HF sequencing datasets from the NCBI Sequence Read Archive (SRA)^2^ were downloaded as SRA files and converted to FASTQ files *via* the SRA toolkit.

### Compilation of gene expression matrices

To obtain high-quality reads, raw data from the GEO dataset were pre-processed using the fastp tool (13), and sample quality was assessed by FastQC and MultiQC (14). The sequences were then aligned against the human reference genome hg38 using STAR (15). Furthermore, the expression values (count matrices) for either gene bodies or called peaks were generated by featureCounts (16).

### Identification of robust differentially expressed genes by the RNA-seq data and robust rank aggregation method

For RNA-seq expression analysis, batch effects were adjusted using the R package RUVSeq, which applies a generalized linear model to regress out the variation estimated from the expression of the housekeeping gene. First, the initial DEGs were detected using the edgeR program package within a single RNA-seq dataset. Second, the RUVg function in RUVSeq was utilized to remove additional sources of unwanted variation (parameter *k* = 1) (17). The remaining non-DEGs were considered as negative control genes and used as housekeeping genes to correct for relative gene expression levels between different samples. Third, based on the corrected gene expression matrix, the corrected DEGs were further obtained by the edgeR package. Fourth, the RRA method-based R package “RobustRankAggreg” was used to integrate the results of RUVSeq analysis of each RNA-seq dataset to identify the final DEGs in patients with HF compared with NFDs. The threshold of DEGs was set as |logFC| > 1, and the significance criterion was set as an adjusted *p*-value < 0.05.

### Functional enrichment analysis

To further investigate the possible functions of DEGs identified by the RUVSeq and the RRA method, GO enrichment and KEGG pathway analyses were performed in the upregulated and downregulated DEGs separately, using the R package “clusterProfiler” (18). The GO term or KEGG pathway with adjusted *p* < 0.05 was considered with significant enrichment. The results were visualized by dot plots using the “dotplot” function of the R package.

### Identification of hub genes

The robust DEG list was uploaded to the Search Tool for the Retrieval of Interacting Genes/Proteins (STRING) database^3^ (19), and the significant protein interaction was determined at the criterion of confidence (combined score) > 0.4. Next, we used Cytoscape software^4^ and cytoHubba (20) plug-in to investigate node composition and pick out hub nodes with a high degree of connectivity in the network.

### Validation of the hub genes using microarray datasets

RNA-seq datasets for HF samples are limited due to a small volume of heart transplant surgeries and the difficulty in obtaining human heart samples. Therefore, in our study, the four eligible HF sequencing datasets (GSE46224, GSE116250, GSE133054, and GSE135055), including 95 HF and NFD samples, were all used for the identification of DEGs, hub genes, and functional enrichment analysis. To further validate the analysis results, HF microarray datasets were acquired from the GEO database. The inclusion criterion was identical to the RUVSeq sources, except that the study type was set as “expression profiling by array.” For the study, four microarray datasets were finally included for the validation: GSE16499 (21), GSE26887 (22), GSE57338 (23), and GSE79962 (24). The gene expression profiling was annotated using the annotation document of corresponding platforms, and the gene expression matrices were column-normalized by the R package “limma” (25) and log-transformed, if necessary. Next, the differential expression of the identified hub genes between patients with HF and NFDs in the microarray datasets was validated and visualized by column graphs.

### Validation of the hub genes using quantitative reverse transcription polymerase chain reaction

For further validation, total RNAs of the heart tissues from patients with HF and NFDs were extracted for the qRT-PCR validation assay. Heart tissues from six patients with HF and eight NFDs were obtained from the Specimen Bank of Cardiovascular Surgery Laboratory and Department of Pathology of Changhai Hospital, Shanghai, China. Written informed consents were obtained from all patients or their family members, and the study was approved by the Institute Ethics Committee of Changhai Hospital.

Total RNAs from the heart tissues were isolated using TRIzol reagent (TRIzol™ Reagent, Invitrogen). RNAs were then reverse-transcribed into cDNAs using a TOYOBO ReverTra Ace^®^ qRT-PCR RT Kit (TOYOBO, Japan). SYBR^®^GREEN (TOYOBO, Japan) was used for qRT-PCR, and the primer sequences used are listed as follows: asporin (*ASPN*) forward, 5′-GGGTGACGGTGTTCCATATC-3′ and reverse, 5′-TTGGCACTGTTGGACAGAAG-3′; collagen type I alpha 1 chain (*COL1A1*) forward, 5′-TCG TGGAAATGATGGTGCTA-3′ and reverse, 5′-ACCAGGTT CACCGCTGTTAC-3′; collagen type IX alpha 2 chain (*COL9A2*) forward, 5′-AAGAGCAACTGGCAGAGGTC-3′ and reverse, 5′-GACCCTCGATCTCCATCCTT-3′; collagen type X alpha 1 chain (*COL10A1*) forward, 5′-TGGGACCCCTC TTGTTAGTG-3′ and reverse, 5′-GCCACACCTGGTCA TTTTCT-3′; cartilage oligomeric matrix protein (*COMP*) forward, 5′-CAGGACGACTTTGATGCAGA-3′ and reverse, 5′-AAGCTGGAGCTGTCCTGGTA-3′; and fibromodulin (*FMOD*) forward, 5′-AGAGAGCTCCAT CTCGACCA-3′ and reverse, 5′-GCAGCTGGTTGT AGGAGAGG-3′. The expression levels of mRNAs relative to glyceraldehyde-3-phosphate dehydrogenase (*GAPDH*) were detected using the 2^–ΔΔ*Ct*^ method.

### Gene set enrichment analysis and gene set variation analysis of the validated hub genes

To further explore potential functions of the hub genes in HF, we performed GSEA and GSVA in the microarray dataset with the maximum HF sample size (GSE57338). The flow of GSEA is as follows: First, correlation analyses were conducted between hub genes and other genes in the gene expression matrix of 54 patients with HF, and genes with the absolute value of correlation coefficient > 0.5 and *p*-value < 0.05 were defined as hub genes-related genes. Then, KEGG pathway enrichment analysis was conducted on these hub genes-related genes using the ClusterProfiler package. For GSVA, 54 patients with HF in the GSE57338 dataset were divided into two groups based on the median expression level of each hub gene (high- and low-expression groups). Then, the ‘‘GSVA’’ package was used to explore the pathways associated with the hub genes. The annotated gene set ‘‘c2.cp.kegg.v7.4.entrez.gmt’’ in the Molecular Signatures Database (MsigDB)^5^ was selected as the reference.

### Construction of the transcription factor–differentially expressed gene regulatory network

It has been reported that binding of TFs to the regulatory regions of genes is a key transcriptional regulatory mechanism to control chromatin and transcription, forming a complex system that guides expression of the genome (26). The TF–DEG regulatory network is constructed by using the following methods: First, the NetworkAnalyst database (27)^6^ and the TF–gene interactions module from the JASPAR database (28) were utilized to explore the possible TFs that could bind to the RRA-identified DEGs. Second, a novel significant TF–DEG regulatory pair was defined in our study according to the following criteria: (i) both the TF and DEG were present in the TF regulatory network constructed by the JASPAR database, and there was predicted interaction between them; (ii) the TF was differentially expressed in patients with HF and NFD samples in the validation set GSE57338 (*p* < 0.05); and (iii) there was a statistically significant relationship between the expression level of TF and its target gene in the validation dataset GSE57338 (the absolute value of correlation coefficient > 0.5 and *p* < 0.05). Third, the constructed TF–DEG regulatory network was visualized using Cytoscape.

### Statistical analysis

Independent two-sample *t*-tests were used to analyze variables with homogeneous variance and normal distribution, whereas Mann–Whitney non-parametric tests were used to analyze variables without homogeneous variance and normal distribution. *P*-values were adjusted for multiple testing by using the Benjamini–Hochberg method. The DEG threshold was set as |logFC| > 1, and the significance criterion was set as an adjusted *p*-value < 0.05. The hypergeometric test was used to calculate the statistical significance of enrichment analysis. An absolute value of the correlation coefficient |r| > 0.3 (*p* < 0.05) indicates a significant interaction relationship (29). All data analyses in the present study were performed by using R (version 3.5.3) and Rstudio (version 1.2.1335). Graphic representations were generated by using GraphPad Prism 9.0 (GraphPad, San Diego, CA, USA) and Cytoscape (Version 3.7.1).

## Results

### Characteristics of the screened heart failure RNA-seq datasets

**FIGURE 1:**
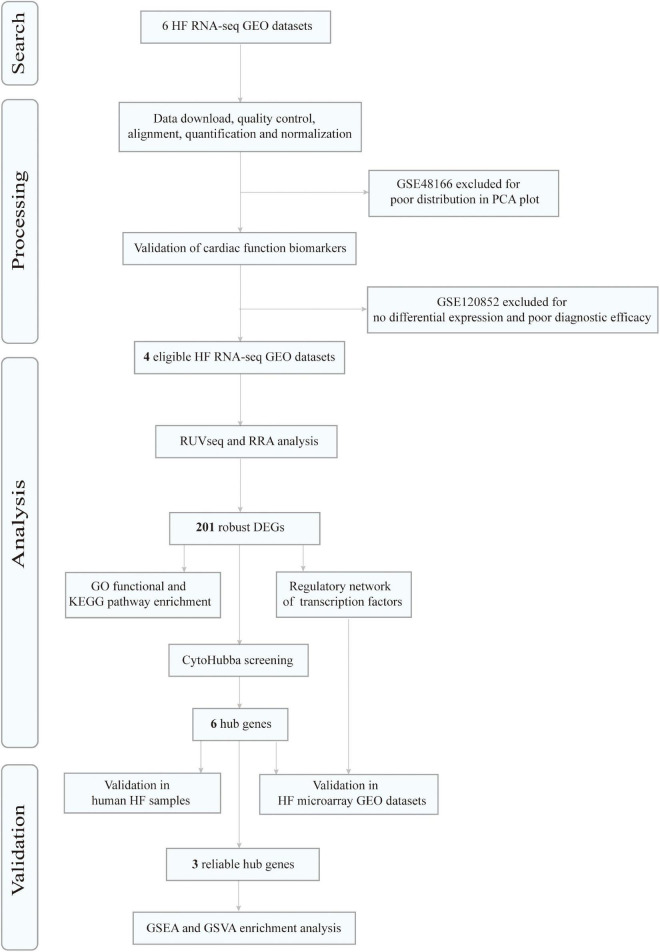
Flowchart of data search, processing, analysis, and validation. HF, heart failure; RNA-seq, RNA sequencing; GEO, Gene Expression Omnibus; PCA, principal component analysis; RRA, robust rank aggregation; DEGs, differentially expressed genes; GO, gene ontology; KEGG, Kyoto Encyclopedia of Genes and Genomes; GSEA, gene set enrichment analysis; GSVA, gene set variation analysis.

Figure 1 depicts the flow diagram of our study. After screening and exclusion according to the aforementioned criteria, six datasets from the GEO database were finally included in this analysis: GSE46224 (30), GSE48166, GSE116250 (31), GSE120852 (32), GSE133054 (33), and GSE135055 (34). The characteristics of these six datasets are summarized in Supplementary Table 1, including the GSE accession number, study country, number of patients with HF and NFDs, and sequencing platform.

### Pre-processing of RNA-seq data

**FIGURE 2:**
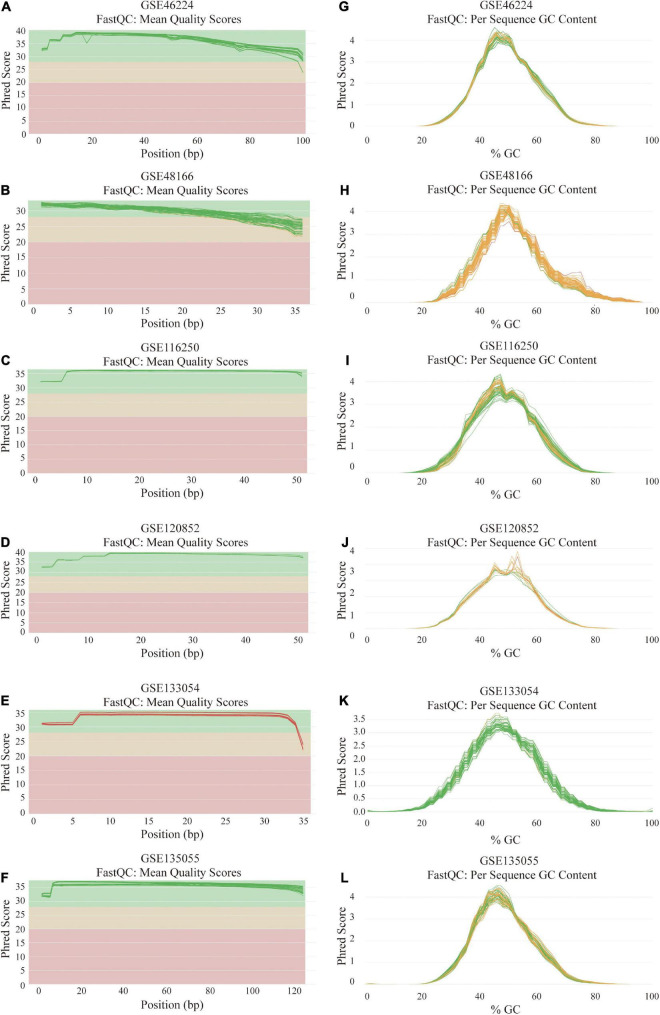
Quality assessment and GC count evaluation of the data from six RNA sequencing datasets. **(A–F)** Per base sequence quality across all samples of GSE46224 **(A)**, GSE48166 **(B)**, GSE116250 **(C)**, GSE120852 **(D)**, GSE133054 **(E)**, and GSE135055 **(F)**. **(G–L)** Per sequence GC content across all samples of GSE46224 **(G)**, GSE48166 **(H)**, GSE116250 **(I)**, GSE120852 **(J)**, GSE133054 **(K)**, and GSE135055 **(L)**.

After the quality-filtering using the fastp tool, the reads with a base quality < 20 or the sequence length ≤ 36 nt were discarded. Then, FastQC was used to assess the sequence quality of the dataset. The final all-in-one quality control report of each dataset was generated using MultiQC. The per base sequence quality and per sequence GC content across all samples of 6 RNA-seq datasets are demonstrated in Figure 2.

### Determination of the selected datasets

**FIGURE 3:**
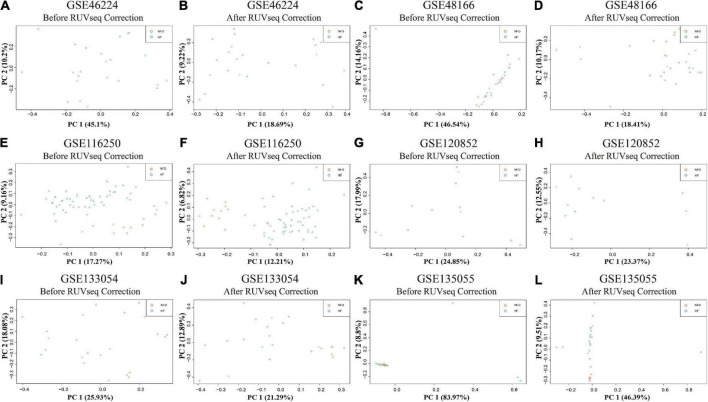
PCA plots of the six RNA-seq datasets in GEO database. PCA distribution plots showed that complete separation between patients with HF and NFD samples was observed in five datasets after RUVSeq correction, except GSE48166 **(C,D)**, namely, GSE46224 **(A,B)**, GSE116250 **(E,F)**, GSE120852 **(G,H)**, GSE133054 **(I,J)**, and GSE135055 **(K,L)**. PCA, principal component analysis; GEO, Gene Expression Omnibus; HF, heart failure; NFD, non-failing donor.

**FIGURE 4:**
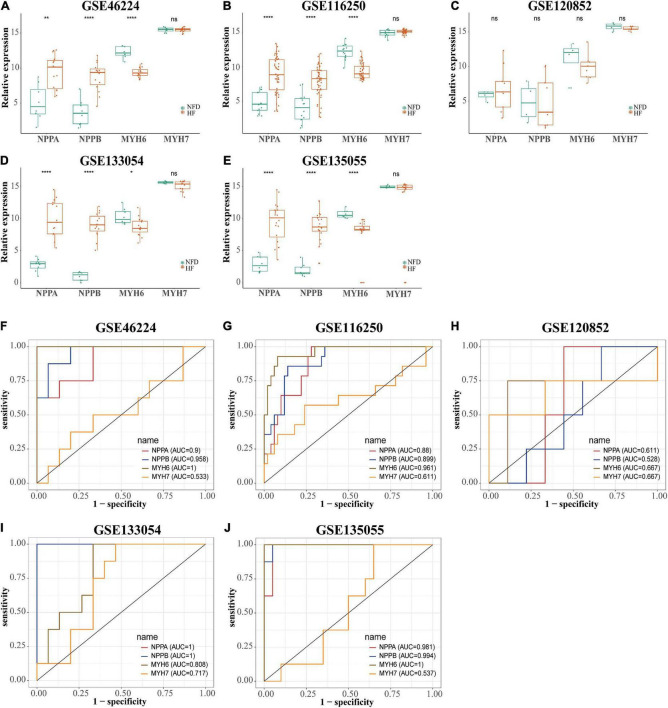
Expression level and diagnostic value of *NPPA*, *NPPB*, *MYH6*, and *MYH7* in the five HF-related RNA-seq datasets. The expression level of *NPPA*, *NPPB*, *MYH6*, and *MYH7* in GSE46224 **(A)**, GSE116250 **(B)**, GSE120852 **(C)**, GSE133054 **(D)**, and GSE135055 **(E)**, respectively. ns, not significant vs. the NFD group; *p < 0.05 vs. the NFD group; **p < 0.01 vs. the NFD group; ****p < 0.0001 vs. the NFD group. The diagnostic values of *NPPA*, *NPPB*, *MYH6*, and *MYH7* in GSE46224 **(F)**, GSE116250 **(G)**, GSE120852 **(H)**, GSE133054 **(I)**, and GSE135055 **(J)**, respectively, as determined by ROC curves. *NPPA*, natriuretic peptide A; *NPPB*, natriuretic peptide B; *MYH6*, myosin heavy chain 6; *MYH7*, myosin heavy chain 7; HF, heart failure; NFD, non-failing donor; ROC, receiver operating characteristic.

Reads were mapped to the human genome (UCSC, hg38) using STAR, and the unique alignments were filtered and presented in Supplementary Table 2. Samples from each dataset were characterized by principal component analysis (PCA) after normalization and adjustment for batch effects using the RUVSeq package. 2D plots of PCA distribution showed that complete separation between samples of patients with HF and NFD samples was observed in five datasets (GSE46224, GSE116250, GSE120852, GSE133054, and GSE135055), except GSE48166 (Figure 3). Hence, dataset GSE48166 was excluded from subsequent analysis. Next, the expression difference and diagnostic efficacy of the four cardiac function markers, namely, natriuretic peptide A (*NPPA*), natriuretic peptide B (*NPPB*), myosin heavy chain 6 (*MYH6*), and myosin heavy chain 7 (*MYH7*), were examined between samples of patients with HF and NFD samples in the five sequencing databases. As shown in Figure 4, the markers showed no differential expression and poor diagnostic performance between the two groups in dataset GSE120852, which was also eliminated from further analysis.

### Identification of robust differentially expressed genes by RNA-seq data and robust rank aggregation method

**FIGURE 5:**
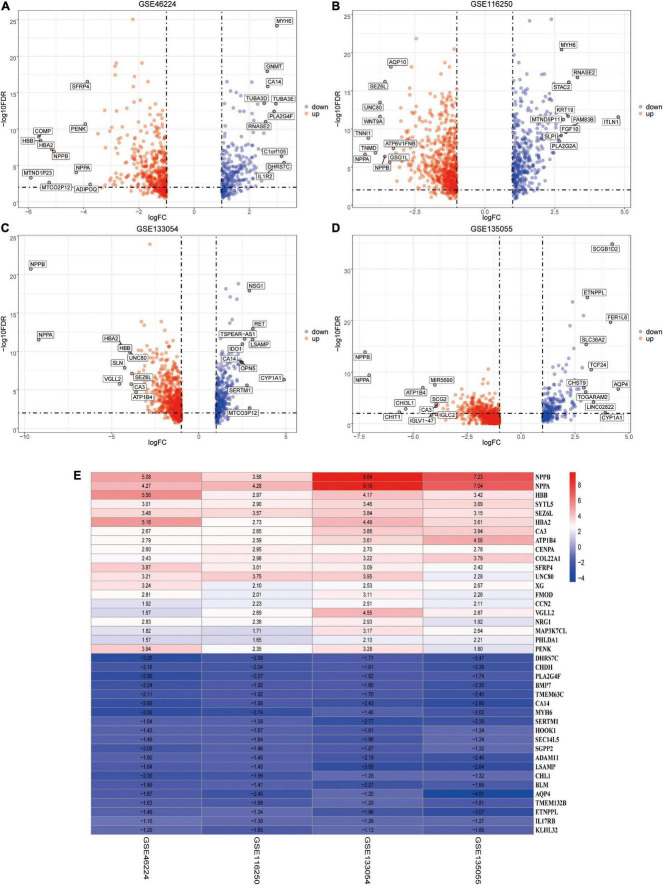
Identification of DEGs by RUVSeq and the RRA method. **(A–D)** Using RUVSeq package, DEGs (patients with HF vs. NFDs) were screened in the four selected RNA-seq datasets GSE46224 **(A)**, GSE116250 **(B)**, GSE133054 **(C)**, and GSE135055 **(D)**, as visualized by volcano plots. Adjusted *p* < 0.05 and |logFC| > 1. **(E)** Heatmap of the top 20 upregulated and the 20 most downregulated DEGs screened from the four selected RNA-seq datasets using RRA analysis. DEGs, differentially expressed genes; RRA, robust rank aggregation; HF, heart failure; NFD, non-failing donor; FC, fold change.

Using the RUVSeq package, DEGs (patients with HF vs. NFDs) were screened for adjusted *p* < 0.05 and |logFC| > 1 in the four identified datasets, respectively, which were visualized by volcano plots (Figures 5A–D). Furthermore, an integrated analysis was performed using the R package “RobustRankAggreg” to generate the differentially expressed ranked gene list. A total of 201 highly ranked DEGs were identified in patients with HF vs. NFD samples, and Supplementary Table 3 exhibits the top 50 upregulated and the top 50 downregulated DEGs. The top 20 upregulated and the 20 most downregulated genes consistently expressed across all datasets were visualized by heatmap, as shown in Figure 5E.

### Functional enrichment analysis of differentially expressed genes

**FIGURE 6:**
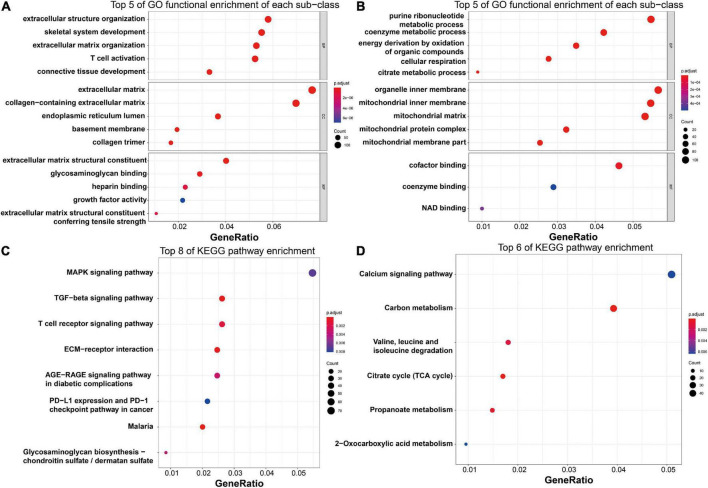
Functional enrichment analysis of the robust HF-related DEGs. **(A)** Top five enriched GO functions of the upregulated genes regarding BP, CC, and MF terms, as visualized by bubble plots. **(B)** Top five enriched GO functions of the downregulated genes regarding BP, CC, and MF terms, as visualized by bubble plots. **(C)** Top eight enriched KEGG pathways of the upregulated genes, as visualized by bubble plots. **(D)** Top eight enriched KEGG pathways of the downregulated genes, as visualized by bubble plots. HF, heart failure; DEGs, differentially expressed genes; GO, gene ontology; BP, biological process; CC, cellular component; MF, molecular function; KEGG, Kyoto Encyclopedia of Genes and Genomes.

To explore the potential biological functions of these DEGs, GO term enrichment and KEGG pathway analyses were performed. The upregulated genes were significantly enriched in extracellular structure organization, skeletal system development, extracellular matrix organization, T-cell activation, and connective tissue development in the biological process (BP) term; the extracellular matrix, collagen-containing extracellular matrix, endoplasmic reticulum lumen, basement membrane, and collagen trimer in the cellular component (CC) term; and extracellular matrix structural constituent, glycosaminoglycan binding, heparin binding, growth factor activity, and extracellular matrix structural constituent conferring tensile strength in the molecular function (MF) term (Figure 6A). For the downregulated genes, the enriched GO functions included purine ribonucleotide metabolic process, coenzyme metabolic process, energy derivation by oxidation of organic compounds, cellular respiration, and citrate metabolic process in the BP category; organelle inner membrane, mitochondrial inner membrane, mitochondrial matrix, mitochondrial protein complex, and mitochondrial membrane part in the CC category; and cofactor binding, coenzyme binding, and NAD binding in the MF category (Figure 6B).

Regarding KEGG pathway analysis, the MAPK signaling pathway, TGF-β signaling pathway, T-cell receptor signaling pathway, Th17 cell differentiation, and ECM–receptor interaction were mostly associated with the upregulated genes (Figure 6C), while the downregulated genes were most enriched in the calcium signaling pathway, carbon metabolism, valine, leucine and isoleucine degradation, citrate cycle, and propanoate metabolism (Figure 6D).

### Hub gene determination

**FIGURE 7:**
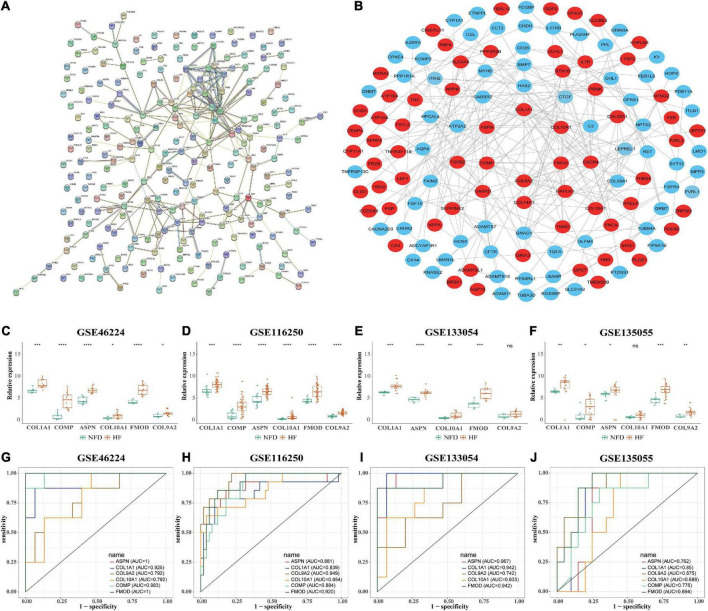
Identification of HF-related hub genes from the four selected RNA-seq datasets. **(A)** PPI network of the 201 DEGs in patients with HF was constructed by using the STRING database. The nodes represent proteins, and the edges represent the interactions. **(B)** Robust hub genes were screened by degree using the cytoHubba plug-in in Cytoscape. The inner the concentric circles, the larger the degree values of the genes. **(C–F)** Relative expression of the six identified hub genes in GSE46224 **(C)**, GSE116250 **(D)**, GSE133054 **(E)**, and GSE135055 **(F)**, respectively. ns, not significant vs. the NFD group; *p < 0.05 vs. the NFD group; **p < 0.01 vs. the NFD group; ***p < 0.001 vs. the NFD group; ****p < 0.0001 vs. the NFD group. **(G–J)** Diagnostic values of the six identified hub genes in GSE46224 **(G)**, GSE116250 **(H)**, GSE133054 **(I)**, and GSE135055 **(J)**, respectively, as determined by ROC curves. HF, heart failure; RNA-seq, RNA sequencing; PPI, protein–protein interaction; DEGs, differentially expressed genes; STRING, search tool for the retrieval of interacting genes; NFD, non-failing donor; ROC, receiver operating characteristic.

The PPI network of the 201 DEGs in patients with HF was constructed by using the STRING database (Figure 7A). Next, the PPI network was loaded into Cytoscape to screen hub genes by degree using the cytoHubba plug-in. As shown in Figure 7B, genes in the inner concentric circles have higher degrees, while genes in the outer concentric circles have relatively lower degrees. Therefore, hub genes were the six genes with the highest degree of connectivity (degree ≥ 10) in the innermost concentric circle: *COL1A1*, *COMP*, *ASPN*, *COL10A1*, *FMOD*, and *COL9A2*.

Furthermore, the relative expression of the identified hub genes in patients with HF and NFD samples was assessed in the four RNA-seq datasets. The results showed that *COL1A1*, *ASPN*, and *FMOD* were consistently upregulated in the HF samples of the four datasets (Figures 7C–F). In addition, univariate ROC analysis was performed to determine the diagnostic accuracy of independent hub genes, suggesting that *COL1A1*, *ASPN*, and *FMOD* had a good diagnostic value in HF (Figures 7G–J).

### Hub gene validation

**FIGURE 8:**
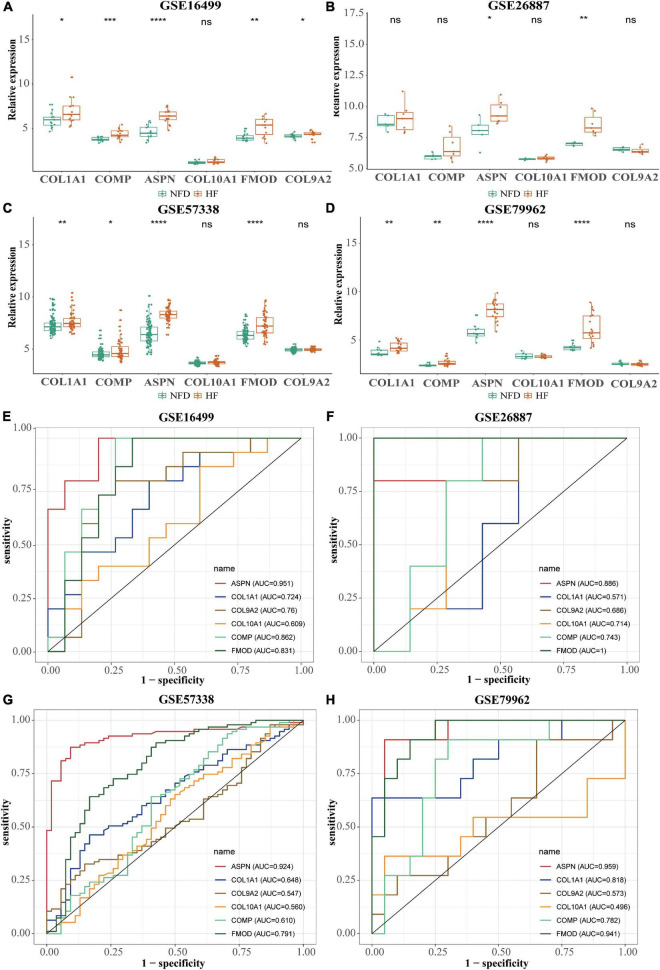
Validation of the six hub genes using four normalized HF-related microarray datasets from the GEO database. **(A–D)** Relative expression of the six hub genes in GSE16499 **(A)**, GSE26887 **(B)**, GSE57338 **(C)**, and GSE79962 **(D)**, respectively. ns, not significant vs. the NFD group; *p < 0.05 vs. the NFD group; **p < 0.01 vs. the NFD group; ***p < 0.001 vs. the NFD group; ****p < 0.0001 vs. the NFD group. **(E–H)** Diagnostic values of the six identified hub genes in GSE16499 **(E)**, GSE26887 **(F)**, GSE57338 **(G)**, and GSE79962 **(H)**, respectively, as determined by ROC curves. HF, heart failure; GEO, Gene Expression Omnibus; NFD, non-failing donor; ROC, receiver operating characteristic.

After normalization, four microarray datasets (GSE16499, GSE26887, GSE57338, and GSE79962) containing human left ventricular samples of HF and NFDs were used to validate the expression of these hub genes (Supplementary Table 4 and Supplementary Figure 1). As shown in Figures 8A–D, the expression of *ASPN* or *FMOD* in HF samples was significantly higher than that in the NFD samples in all four datasets, and *COL1A1* or *COMP* showed the similar upregulation in three datasets. However, the expression of *COL9A2* or *COL10A1* was not statistically different in the HF and NFD samples in these datasets. Consistently, the diagnostic values of the hub genes suggested by the ROC curves revealed the same trend (Figures 8E–H).

**FIGURE 9:**
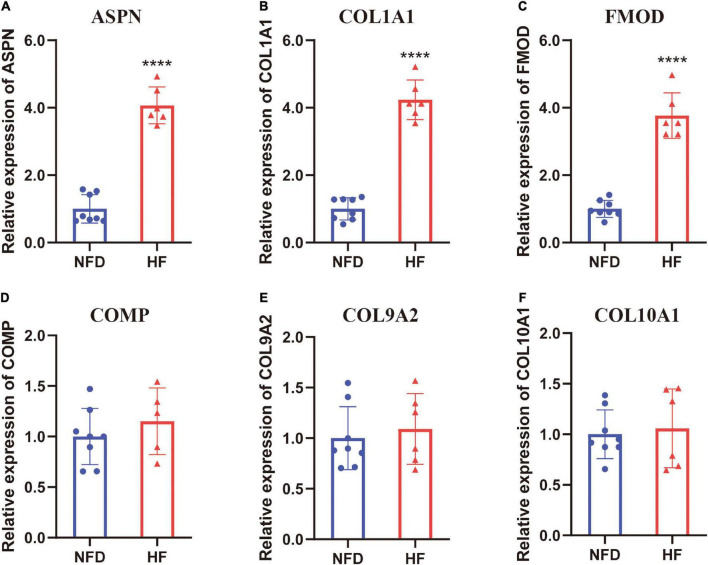
Validation of the six hub genes by qRT-PCR using human heart tissues from patients with HF and NFDs. The expression level of *ASPN* **(A)**, *COL1A1* **(B)**, *FMOD* **(C)**, *COMP* **(D)**, *COL9A2* **(E)**, and *COL10A1* **(F)** in heart tissues from HF patients and NFDs, as determined by qRT-PCR. Data are presented with mean ± SD. ****p < 0.0001 vs. the NFD group. QRT-PCR, quantitative real-time reverse transcription PCR; HF, heart failure; NFD, non-failing donor; SD, standard deviation.

In addition to the microarray datasets, the expression of hub genes was further validated by qRT-PCR experiments using 14 heart tissues from patients with HF or NFDs. As described in Figure 9, *ASPN*, *COL1A1*, and *FMOD* were significantly upregulated in the six heart tissues of patients with HF compared with NFDs. Taken together, these validation results confirmed the differential expression and diagnostic value of *ASPN*, *COL1A1*, and *FMOD* as reliable hub genes in HF development.

### Gene set enrichment analysis and gene set variation analysis reveal a close relationship between hub genes and glucose metabolism-related pathways

**FIGURE 10:**
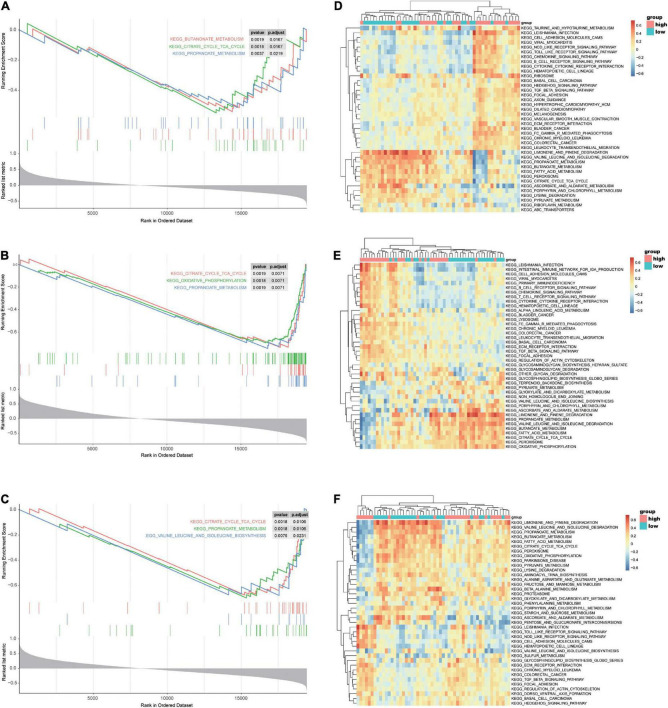
GSEA and GSVA of *ASPN*, *COL1A1*, and *FMOD* in the selected microarray dataset GSE57338. **(A–C)** GSEA-enriched pathways of DEGs related to *ASPN* **(A)**, *COL1A1* **(B)**, or *FMOD* **(C)** expression in the GSE57338 dataset. **(D–F)** GSVA-derived clustering heatmaps showing the enriched pathways of DEGs related to *ASPN* **(D)**, *COL1A1* **(E)**, or *FMOD* **(F)** expression in GSE57338 dataset. GSEA, gene set enrichment analysis; GSVA, gene set variation analysis.

To reveal the underlying mechanism of the three reliable hub genes (*ASPN*, *COL1A1*, and *FMOD*) involved in HF, GSEA was conducted to explore significantly enriched pathways associated with the hub genes in the validation dataset GSE57338. As shown in Figures 10A–C, the top three signaling pathways enriched by the DEGs between subgroups were identified, among which citrate cycle (TCA cycle) and propanoate metabolism pathways were significantly enriched in the subgroups of all the three hub genes. In addition, the enrichment in glucose metabolism-related pathways was further confirmed by GSVA (Figures 10D–F).

### Identification of signatures of transcription factor–differentially expressed gene regulatory network

**FIGURE 11:**
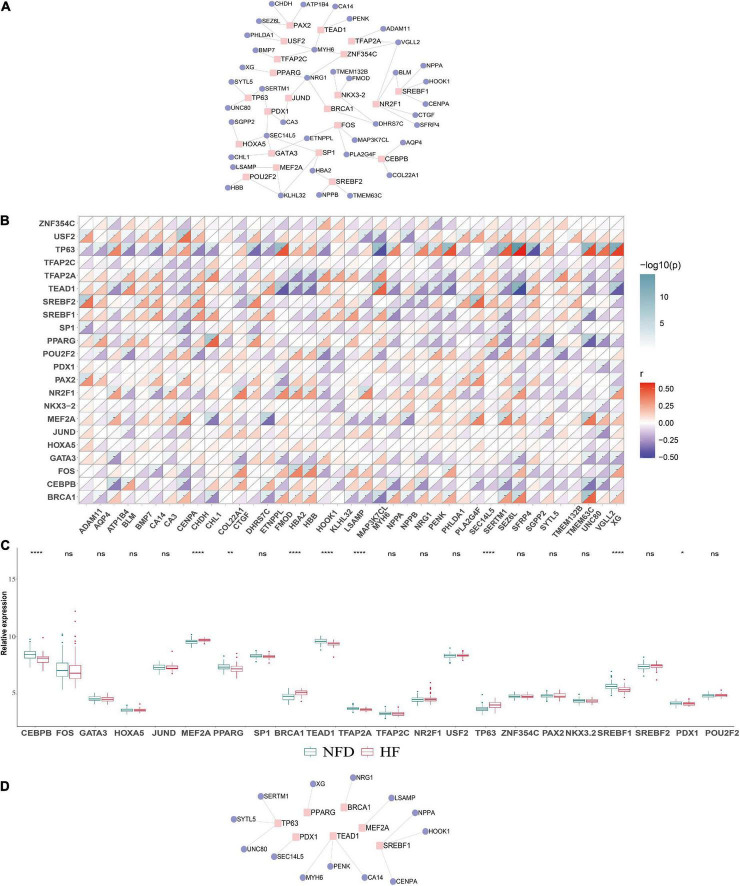
Construction of the TF–DEGs regulatory network in HF. **(A)** TF–DEG regulatory network was established based on the top 20 upregulated and the 20 most downregulated DEGs. **(B)** Correlation matrix between the identified TFs and DEGs. An absolute value of correlation coefficient |r| > 0.3 and *p* < 0.05 indicates a statistically significant relationship. **(C)** Relative expression of the identified TFs in the validation microarray dataset GSE57338. ns, not significant vs. the NFD group; *p < 0.05 vs. the NFD group; **p < 0.01 vs. the NFD group; ****p < 0.0001 vs. the NFD group. **(D)** Identified significant TF–DEG regulation pairs according to the criteria. TFs, transcription factors; DEGs, differentially expressed genes; HF, heart failure; NFD, non-failing donor.

To determine the potential roles of TFs in regulating the transcriptional expression of DEGs, the specific TF–gene regulatory network was established based on the top 20 upregulated and the 20 most downregulated integrated DEGs (Figure 11A). As demonstrated in Figures 11B,C, several TFs, including CEBPB, MEF2A, PPARG, BRCA1, TEAD1, TFAP2A, TP63, SREBF1, and PDX1, showed significant correlation with multiple DEGs and were differentially expressed in patients with HF and NFDs in GSE57338 (*p* < 0.05). According to the defining criteria of the significant TF–DEG regulation pair, we identified TP63-*SERTM1*/*SYTL5*/*UNC80*, PPARG-*XG*, BRCA1-*NRG1*, MEF2A-*LSAMP*, SREBF1-*NPPA*/*HOOK1*/*CENPA*, TEAD1-*CA14*/*MYH6*/*PENK*, and PDX1-*SEC14L5* as significant TF–DEG pairs (Figure 11D).

## Discussion

In the present study, four HF RNA-seq GEO datasets (GSE46224, GSE116250, GSE133054, and GSE135055) were finally included, involving a total of 100 patients with HF and 38 NFDs. In total, 201 robust HF-related DEGs were obtained utilizing RUVSeq and RRA method, and *ASPN*, *COL1A1*, *COL9A2*, *COL10A1*, *COMP*, and *FMOD* were identified as hub genes with the highest degree of connectivity using STRING database and cytoHubba plug-in. Among them, *ASPN*, *COL1A1*, and *FMOD* exhibited differential expressions and excellent diagnostic efficiency in all four RNA-seq datasets, which were further validated using data from the four screened HF microarray datasets (GSE16499, GSE26887, GSE57338, and GSE79962). Moreover, the significant upregulation of *ASPN*, *COL1A1*, and *FMOD* was experimentally confirmed by qRT-PCR using the heart tissues of patients with HF and NFD samples. In addition, functional enrichment analysis showed that myocardial fibrosis-related pathways resulted from T-cell-mediated immune response and myocardial glucose metabolism were closely associated with the onset and progression of HF. In addition to this, the TF–DEG regulatory network was established, and 13 significant TF–DEG pairs were identified.

Despite the great advancement in HF medical treatment, it remains the major and growing public health problem that leads to considerable morbidity and mortality (35). Robust biomarkers for early diagnosis of HF are the key for novel targeted pharmacological approaches and for improving the prognosis of patients (36). Consistently, serum type B natriuretic peptide (*BNP*) has been recognized as a well-established biomarker for the diagnosis of HF. However, a recent study reported that a subset (4.9%) of hospitalized patients with confirmed HF had unexpectedly low BNP levels (<50 pg/ml), and a small proportion (0.1–1.1%) had BNP levels even below detection limits (37). Therefore, it remains urgent to explore novel molecules with potentially new mechanisms for the development of HF.

Recently, gene mining using microarrays or RNA-seq datasets has been widely used to generate the transcriptomic profiles of HF development. Zhang et al. identified six hub genes (*BCL3*, *HCK*, *PPIF*, *S100A9*, *SERPINA1*, and *TBC1D9B*) as potential biomarkers of HF by using the weighted gene co-expression network analysis (WGCNA) method through three HF datasets, namely, GSE59867, GSE1869, and GSE42955 (38). Tian et al. constructed a random forest algorithm and artificial neural network and detected six hub genes by mining of two HF datasets (GSE57345 and GSE141910) (39). However, the aforementioned studies were based on the integration of DEGs, rather than raw data from different datasets. To date, the inconsistency between different platforms and datasets remains the major hurdle blocking the bioinformatics mining of HF-related genes, especially for RNA-seq datasets.

The RRA method, a recently emerging analysis method, has been widely used to integrate different datasets and produce a ranked list of the DEGs (40). For example, Ma et al. utilized the RRA method to integrate four eligible DCM microarray datasets from the GEO and developed a 7-gene signature predictive model of DCM (11). While in the present study, using RUVSeq to substantially decrease batch effects, we integrated, for the first time, the different RNA-seq datasets of the GEO database to explore DEGs and hub genes associated with HF by using the RRA method. Through internal RNA-seq dataset and external microarray dataset validation, *ASPN*, *COL1A1*, and *FMOD* were finally identified as real hub genes of HF, which were further confirmed by qRT-PCR using the heart tissues from patients with HF and NFDs.

Interestingly, the identified hub genes *ASPN* (41), *COL1A1* (42), and *FMOD* (43), all belong to the type I collagen members in the extracellular matrix (ECM) composition and have been reported to play important roles in the development and progression of various diseases, especially malignant tumors. For example, *ASPN* was reported to enhance tumor invasion and cancer-associated fibroblasts *via* activation of the CD44-Rac1 pathway in gastric cancer (41). Ma et al. highlighted the role of *COL1A1* as a potential diagnostic biomarker and therapeutic target in early development and metastasis of hepatocellular carcinoma (42). Ao et al. revealed that *FMOD* could promote tumor angiogenesis by upregulating the expression of angiogenic factors in human small-cell lung cancer (43). Regarding the function of hub genes in HF development, a multi-level transcriptomic study conducted by Hua et al. suggested that *COL1A1* might be a plasma biomarker of HF and associated with HF progression, especially to predict the 1-year survival from HF onset to transplantation. A *COL1A1* content ≥ 256.5ng/ml in plasma was found to be associated with poor survival within 1 year of heart transplantation from HF (34). In the study conducted by Andenæs et al., *FMOD* was found 3–10-fold upregulated in hearts of patients with HF and mice, and *FMOD*-KO mice showed a relatively mild hypertrophic phenotype (44). However, to the best of our knowledge, there are no experimental studies focusing on the role of *ASPN* in HF. Therefore, our multi-dataset RRA analysis, followed by microarray dataset and experimental validation, provides more robust and comprehensive evidence for the value of the three ECM-related genes, namely, *COL1A1*, *FMOD*, and *ASPN*, in HF development.

Recent advances have highlighted the crucial role of immune activation in the development and progression of HF. A study by Aghajanian et al. demonstrated that adoptive transfer of T cells that express a chimeric antigen receptor against fibroblast activation protein can inhibit myocardial fibrosis and improve cardiac function in mice (45). Consistently, according to the GO term analysis in our study, the upregulated HF-related DEGs were enriched in T-cell activation of the “BP” term, the extracellular matrix of “CC” terms, and the extracellular matrix structural constituent of “MF” terms. Moreover, regarding the KEGG pathway analysis, the T-cell receptor signaling pathway and ECM–receptor interaction were identified as the significantly enriched pathways of the upregulated DEGs. Considering that all the three hub genes—*ASPN*, *COL1A1*, and *FMOD*—are closely associated with the ECM, we thus speculate a potentially key pathway in the development of HF, that is, T-cell-mediated immune responses lead to the imbalance in ECM anabolism and catabolism, ultimately resulting in myocardial fibrosis and HF.

To further explore the potential mechanism of *ASPN*, *COL1A1*, and *FMOD* in HF, we performed GSEA and GSVA on the validation dataset of GSE57338. Results showed that *ASPN*-, *COL1A1*-, or *FMOD*-related DEGs were enriched in the “citric acid cycle (TCA cycle)” and “propionic acid metabolism” pathways, both of which are closely associated with glucose metabolism (46, 47). Notably, targeting cardiac glucose metabolism has been recognized as a promising therapeutic strategy for HF treatment. Liu et al. reported that dichloroacetate, a pyruvate dehydrogenase kinase inhibitor, could alter glucose metabolism in cardiomyocytes by stimulating the activity of pyruvate dehydrogenase complex, thereby improving cardiac efficiency (48). In addition, inhibitors of fatty acid oxidation such as trimetazidine (49), perhexiline (50), and etomoxir (51) can improve cardiac function in patients with HF by increasing glucose oxidation.

Aberrant regulation of TFs is strongly associated with the onset and progression of HF (52). Therefore, in our research, we further investigated the TF–gene interactions to detect the transcriptional regulators of the robust DEGs. Among the seven identified significant TFs, MEF2A (53) and PPARG (54) have been reported to play a role in cardiac remodeling and water retention in HF, respectively. Liu et al. found that suppressing expression of TEAD1, the Hippo signaling effector, could activate the necroptotic pathway and induce massive cardiomyocyte necroptosis, ultimately leading to impaired cardiac function (55). Moreover, loss of BRCA1 in mouse cardiomyocytes resulted in adverse cardiac remodeling and poor ventricular function (56). Although the functions of these TFs in HF have been partially reported, the regulatory relationship of the TF–DEG pairs and the in-depth molecular mechanisms remain to be further validated through HF-related experimental studies.

Our study has several limitations. First, the sample size of patients with HF is relatively small, although we have included as many datasets that met the criteria as possible. Future studies with larger sample sizes are needed to confirm these findings. Second, this study is mainly based on bioinformatics analysis and qRT-PCR validation of hub gene expression. Further experimental research is needed to clarify the in-depth mechanism of the hub gene-related HF regulation. Third, information about disease grades, treatment methods, and prognosis of patients with HF is not available in the database, leading to the failure to analyze correlation between hub genes and clinical characteristics or prognosis of HF. Fourth, the etiology of HF is complex, involving multiple environmental factors in addition to genetic factors (57), such as behavioral factors, socioeconomic and psychosocial factors, air quality, and meteorological factors (58–60). Horton et al. reported that the influence of modifiable lifestyle factors cannot be ignored in the development of direct-to-consumer (DTC) genetic tests (61). In recent years, emerging evidence has shown that gene–environment interactions play an important role in complex disease progression. Bentley et al. revealed that the genetic associations with lipids could be modified by smoking (62). Therefore, future research needs to further focus on the role of environmental factors and gene–environment interactions in HF.

## Conclusion

In conclusion, the present study integrated, for the first time, the different RNA-seq datasets of HF from the GEO database and identified robust HF-related DEGs utilizing RUVSeq and the RRA method. Furthermore, three reliable hub genes—*ASPN*, *COL1A1*, and *FMOD*—were screened and validated by bioinformatics and experimental assays. Functional enrichment analysis showed that DEGs and hub genes were associated with T-cell-mediated immune response and the glucose metabolism signaling pathway. In addition, significant TF–DEG regulatory network of HF was further established. However, high-quality basic or clinical research is required to deeply investigate the mechanisms by which these hub genes are involved in HF and to confirm their values as biomarker for HF diagnosis and treatment.

## Data availability statement

The datasets presented in this study can be found in online repositories. The names of the repository/repositories and accession number(s) can be found in the article/Supplementary material.

## Ethics statement

The studies involving human participants were reviewed and approved by the Institute Ethics Committee of Changhai Hospital. The patients/participants provided their written informed consent to participate in this study.

## Author contributions

ZG, XS, and XZ contributed to the conception of the study. DT and CM performed the study execution and experiments. ZZ and QX contributed to part of bioinformatic analysis. DT, CM, and XS prepared the manuscript. XZ and ZG contributed to the funding of the study. All authors reviewed the manuscript, provided critical revision, and have approved the final version for publication.

## Footnotes

1
www.ncbi.nlm.nih.gov/geo/

2
https://www.ncbi.nlm.nih.gov/sra/

3
https://string-db.org/

4
www.Cytoscape.org/

5
http://www.gsea-msigdb.org/gsea/downloads.jsp

6
https://www.networkanalyst.ca/
