# Supplementary material for: Identification of hub genes and transcription factor regulatory network for heart failure using RNA-seq data and robust rank aggregation analysis
Source: Front Cardiovasc Med. 2022 Oct 28;9:916429. doi: 10.3389/fcvm.2022.916429 (PMC9649652; doi:10.3389/fcvm.2022.916429)
Supplement: Supplementary file 1 [file Data_Sheet_1.PDF]

Supplementary Material for

Identification of hub genes as potential biomarkers for heart failure using RUVseq and robust rank aggregation analysis

Table S1. The characteristics of the 6 screened RNA-seq datasets of HF in GEO database

Table S2. The mapping results of the 6 HF RNA-seq datasets to human genome (UCSC, hg38)

Table S3. The list of the top 100 significant DEGs identified using RUV-seq and RRA

Table S4. The characteristics of the 4 validation microarray datasets of HF in GEO database

Figure S1. The column-normalization of gene expression matrices in the 4 validation microarray datasets of HF

References

Abbreviations: RNA-seq, RNA-sequencing; HF, heart failure; NFD, nonfailing donors; GEO, Gene Expression Omnibus; RRA, robust rank aggregation;

Table S1. The characteristics of the 6 screened RNA-seq datasets of HF in GEO database

Dataset ID	Country	Number of samples		Sequencing platform
		HF	NFD	
GSE46224[(1)]	USA	15	8	Illumina HiSeq 2000
GSE48166	USA	15	15	Illumina Genome Analyzer II
GSE116250[(2)]	USA	50	14	Illumina HiSeq 2500
GSE120852[(3)]	USA	10	5	Illumina HiSeq 2500
GSE133054[(4)]	USA	15	8	Illumina NextSeq 500
GSE135055[(5)]	China	15	15	Illumina Genome Analyzer II

Table S2. The mapping results of the 6 HF RNA-seq datasets to human genome (UCSC, hg38)

Dataset ID	Total reads	Average uniquely mapped reads	Average uniquely mapped rate	Average multi-mapped reads	Average multi-mapped rate
GSE46224	13972421±4296694	13063999±4001157	93.54%±0.78%	832762±288489	5.92%±0.79%
GSE48166	14272079±2692650	4935721±2009705	32.52%±5.39%	9070038±1804135	63.57%±4.74%
GSE116250	47976875±6080451	39475490±4948439	82.30%±1.26%	7760132±1429978	16.11%±1.58%
GSE120852	12979230±2121082	9457371±1364380	73.21%±4.75%	2375833±389129	18.37%±1.71%
GSE133054	21766305±4693337	17550617±3688579	80.76%±1.61%	2755090±823537	12.50%±1.70%
GSE135055	42078269±2738112	39489984±2545203	93.85%±0.49%	2136658±241989	5.07%±0.39%

Table S3. The list of the top 50 upregulated and the 50 most downregulated DEGs identified using RUV-seq and RRA method

Name	Pvalue	AdjPvalue	LogFC	DiffState
NPPB	3.79E-14	7.29E-10	6.38139403	up
NPPA	7.03E-14	1.35E-09	6.19605153	up
HBB	3.07E-12	5.91E-08	4.02840747	up
SYTL5	4.68E-12	0.00000009	3.26438445	up
SEZ6L	5.69E-12	1.09E-07	3.5091206	up
HBA2	1.14E-11	0.00000022	3.99583531	up
CA3	3.47E-11	6.67E-07	3.28712357	up
ATP1B4	6.77E-11	0.0000013	3.38596962	up
CENPA	7.49E-11	0.00000144	2.75944887	up
COL22A1	9.11E-11	0.00000175	3.10584356	up
SFRP4	3.31E-10	0.00000637	3.09746326	up
UNC80	1.08E-09	0.0000208	3.29537165	up
XG	1.14E-09	0.0000219	2.63499144	up
FMOD	1.92E-09	0.0000369	2.54602106	up
CCN2	4.44E-09	0.0000854	2.19237725	up
VGLL2	9.16E-09	0.00017606	2.94657008	up
NRG1	1.37E-08	0.00026274	2.50848093	up
MAP3K7CL	1.65E-08	0.00031648	2.33482995	up
PHLDA1	2.39E-08	0.00045899	1.88401664	up
PENK	3.07E-08	0.00059067	2.84507701	up
CHD5	3.5E-08	0.00067346	2.2534224	up
AEBP1	3.98E-08	0.00076465	2.03743274	up
TNMD	4.43E-08	0.00085091	2.59571372	up
ZNF365	4.87E-08	0.00093684	2.29755985	up
SLITRK4	6.14E-08	0.00118008	1.97089199	up
CRACD	6.7E-08	0.00128769	2.16388515	up

ANKRD34C	8.19E-08	0.0015746	1.91205137	up
HAPLN1	9.23E-08	0.00177522	2.29207924	up
RASL11B	1.18E-07	0.00226913	1.91428539	up
ATP1A4	1.49E-07	0.00285958	1.65627209	up
TNFRSF11B	1.63E-07	0.00312542	1.62205978	up
UCHL1	1.77E-07	0.00340937	2.30163719	up
GDF6	1.85E-07	0.00355839	2.0589248	up
ALKAL2	1.93E-07	0.00371225	1.95308042	up
P3H2	0.00000026	0.00499817	1.69027139	up
SEZ6L2	0.00000027	0.00519642	1.60270968	up
TGFB2	3.07E-07	0.00590008	1.6721457	up
PPP2R2B	3.31E-07	0.00635518	2.14407799	up
NXPH4	3.82E-07	0.00734386	1.48200132	up
THBS4	4.75E-07	0.00913967	1.56802299	up
AQP10	4.92E-07	0.00945078	2.25538035	up
C16orf89	4.97E-07	0.00955623	1.57482287	up
TMEM30B	5.25E-07	0.01009676	1.66142921	up
SERPINE2	5.35E-07	0.01028905	1.94122879	up
DNAJC22	0.00000063	0.01210745	1.69469109	up
FRZB	6.63E-07	0.01275218	1.67851485	up
FAP	0.00000067	0.01288416	1.97930264	up
PDE8B	7.34E-07	0.01411855	1.60317137	up
LEFTY2	7.37E-07	0.01417845	2.05904637	up
DLGAP1	7.49E-07	0.01440448	2.101813	up
DHRS7C	1.98E-10	0.00000381	-2.38297125	down
CHDH	2.14E-10	0.00000411	-2.09786133	down
PLA2G4F	4.61E-10	0.00000886	-2.15452143	down
BMP7	5.55E-10	0.0000107	-2.06663659	down
TMEM63C	5.9E-10	0.0000113	-2.04277001	down

CA14	8.78E-10	0.0000169	-2.46674527	down
MYH6	4.13E-09	0.0000794	-2.29186985	down
SERTM1	4.44E-09	0.0000854	-2.09217474	down
HOOK1	8.36E-09	0.00016071	-1.58847396	down
SEC14L5	8.62E-09	0.00016571	-1.60681469	down
SGPP2	1.12E-08	0.00021616	-1.68295742	down
ADAM11	1.19E-08	0.00022878	-1.92420407	down
LSAMP	1.37E-08	0.00026274	-2.30428554	down
CHL1	1.82E-08	0.00035066	-1.72044024	down
BLM	2.17E-08	0.00041706	-1.85805039	down
AQP4	3.14E-08	0.00060391	-2.50860812	down
TMEM132B	3.35E-08	0.00064495	-1.63351442	down
ETNPPL	3.43E-08	0.00065909	-1.96003322	down
IL17RB	5.48E-08	0.0010532	-1.23114174	down
KLHL32	7.11E-08	0.00136669	-1.45883287	down
GNMT	8.92E-08	0.00171605	-1.74024881	down
MEI4	9.87E-08	0.00189813	-1.70338955	down
FAIM2	1.09E-07	0.00209433	-1.28133189	down
FAM81A	1.14E-07	0.00219795	-1.24531997	down
CADPS2	1.26E-07	0.00241667	-1.16979754	down
TNFRSF13C	1.51E-07	0.00290266	-1.57743356	down
NSG1	1.55E-07	0.00297789	-2.04147657	down
TCF24	1.82E-07	0.00350819	-1.8853291	down
TOGARAM2	2.04E-07	0.00392509	-1.83223996	down
IL20RA	2.07E-07	0.00397969	-1.23569991	down
HOPX	2.34E-07	0.0044972	-1.66472865	down
CPNE4	2.84E-07	0.00546986	-1.28221657	down
SYT13	4.24E-07	0.00815785	-1.32899457	down
GBX1	4.44E-07	0.00854056	-1.38273331	down

TUBB4A	4.97E-07	0.00955623	-1.47515336	down
CACNA2D3	0.00000061	0.01173254	-1.07013278	down
ADH1B	6.77E-07	0.01301716	-1.12214981	down
RNF157	7.05E-07	0.01355946	-1.114391	down
C3	7.49E-07	0.01440448	-1.11220029	down
TMEM178B	7.64E-07	0.01469474	-1.11085096	down
PTDSS1	9.11E-07	0.01750874	-1.0212589	down
ART5	0.00000101	0.01941631	-1.2002502	down
CYP1A1	0.00000104	0.01996534	-2.20243389	down
KCNIP2	0.00000109	0.02089853	-1.35227039	down
GRB7	0.00000128	0.02454271	-1.19974284	down
OPN5	0.00000134	0.02585721	-1.58001023	down
CHAC2	0.00000139	0.02676241	-1.03404267	down
HERC2P3	0.00000139	0.02676241	-1.11612551	down
LMAN1L	0.00000153	0.02939047	-1.53809119	down
TGFA	0.00000157	0.03011882	-1.21450261	down

Table S4. The characteristics of the 4 validation microarray datasets of HF in GEO database

Dataset ID	Country	Number of samples		Platform ID
		HF	NFD	
GSE16499(6)	USA	15	15	GPL5175
GSE26887(7)	Italy	7	5	GPL6244
GSE57338(8)	USA	54	95	GPL11532
GSE79962(9)	USA	20	11	GPL6244

Figure S1. The column-normalization of gene expression matrices in the 4 validation microarray datasets of HF

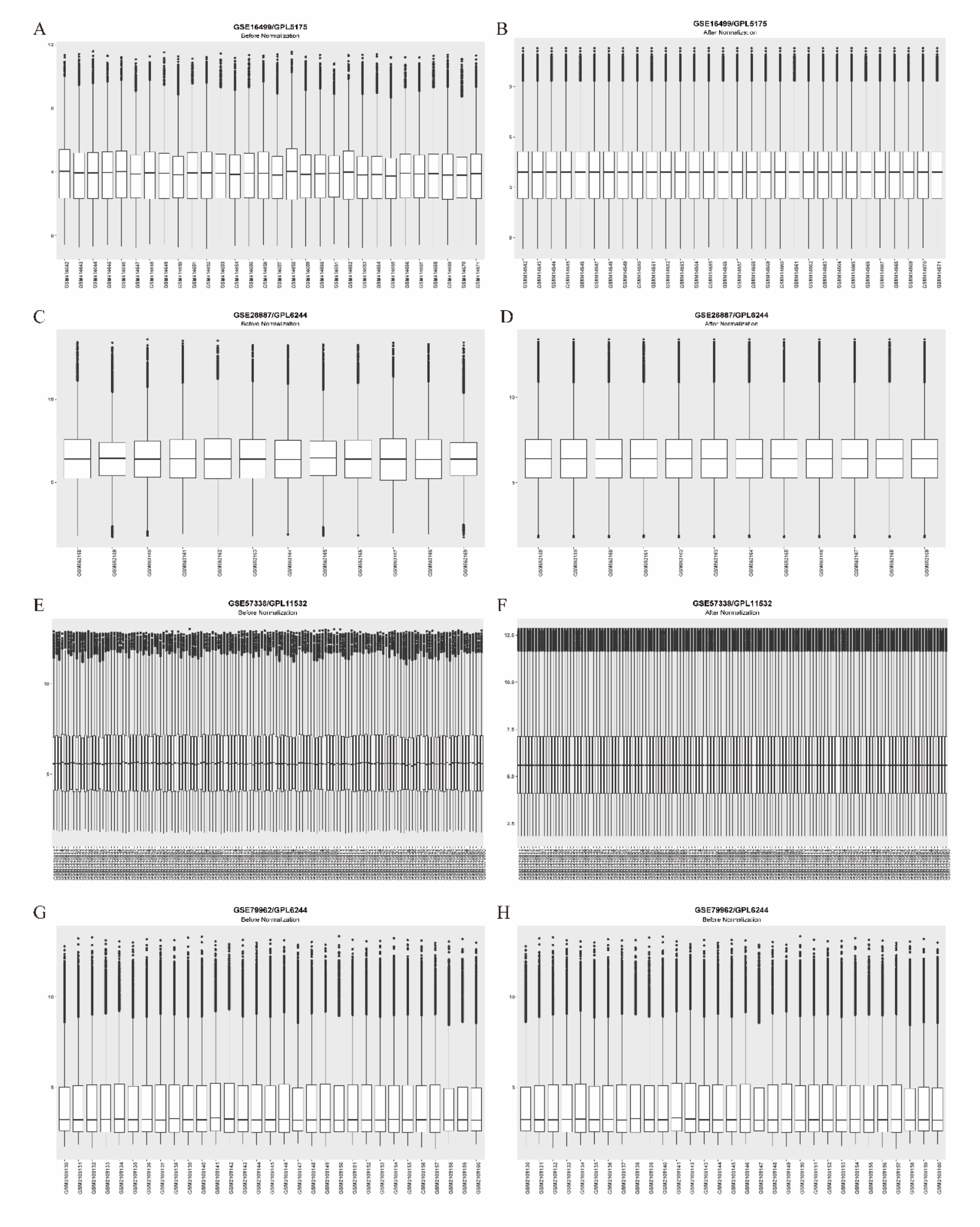

The gene expression profiling was annotated using the annotation document of corresponding platforms, and the gene expression matrices were column-normalized by the R package "limma". Before normalization: GSE16499 (A), GSE26887 (C), GSE57338 (E) and GSE79962 (G); after normalization: GSE16499 (B), GSE26887 (D), GSE57338 (E) and GSE79962 (F).

References

1. Yang KC, Yamada KA, Patel AY, Topkara VK, George I, Cheema FH, et al. Deep RNA sequencing reveals dynamic regulation of myocardial noncoding RNAs in failing human heart and remodeling with mechanical circulatory support. Circulation. 2014;129(9):1009-21.
2. Sweet ME, Cocciolo A, Slavov D, Jones KL, Sweet JR, Graw SL, et al. Transcriptome analysis of human heart failure reveals dysregulated cell adhesion in dilated cardiomyopathy and activated immune pathways in ischemic heart failure. BMC Genomics. 2018;19(1):812.
3. Tzimas C, Rau CD, Buergisser PE, Jean-Louis G, Jr., Lee K, Chukwuneke J, et al. WIPI1 is a conserved mediator of right ventricular failure. JCI Insight. 2019;5(11).
4. Ren Z, Yu P, Li D, Li Z, Liao Y, Wang Y, et al. Single-Cell Reconstruction of Progression Trajectory Reveals Intervention Principles in Pathological Cardiac Hypertrophy. Circulation. 2020;141(21):1704-19.
5. Hua X, Wang YY, Jia P, Xiong Q, Hu Y, Chang Y, et al. Multi-level transcriptome sequencing identifies COL1A1 as a candidate marker in human heart failure progression. BMC Med. 2020;18(1):2.
6. Kong SW, Hu YW, Ho JW, Ikeda S, Polster S, John R, et al. Heart failure-associated changes in RNA splicing of sarcomere genes. Circ Cardiovasc Genet. 2010;3(2):138-46.
7. Greco S, Fasanaro P, Castelvecchio S, D'Alessandra Y, Arcelli D, Di Donato M, et al. MicroRNA dysregulation in diabetic ischemic heart failure patients. Diabetes. 2012;61(6):1633-41.
8. Liu Y, Morley M, Brandimarto J, Hannenhalli S, Hu Y, Ashley EA, et al. RNA-Seq identifies novel myocardial gene expression signatures of heart failure. Genomics. 2015;105(2):83-9.
9. Matkovich SJ, Al Khiami B, Efimov IR, Evans S, Vader J, Jain A, et al. Widespread Down-Regulation of Cardiac Mitochondrial and Sarcomeric Genes in Patients With Sepsis. Crit Care Med. 2017;45(3):407-14.
